# Restoration of gut dysbiosis through *Clostridium butyricum* and magnesium possibly balance blood glucose levels: an experimental study

**DOI:** 10.1186/s12866-024-03218-3

**Published:** 2024-04-01

**Authors:** Hafiz Muhammad Ubaid Tayyib, Amjed Ali, Shaista Jabeen, Habib-ur-Rehman, Hafsa Kamran, Majed A. Bajaber, Muhammad Usman, Xiao Zhang

**Affiliations:** 1 Department of Bioinformatics, School of Medical Informatics and Engineering, Xuzhou Medical University, Xuzhou, P. R. China; 2 University Institute of Diet and Nutritional Sciences, Faculty of Allied Health Sciences, The University of Lahore, Lahore, Pakistan; 3 University Institute of Physical therapy, Faculty of Allied Health Sciences, The University of Lahore, Lahore, Pakistan; 4 Department of Chemistry, Faculty of Science, King Khalid University, P.O. Box 9004, Abha, 61413 Saudi Arabia; 5 Yantai Longch Technologies. CO., LTD, Yantai, P. R. China

**Keywords:** *Clostridium butyricum*, Diabetes Mellitus, Magnesium supplementation, Probiotic

## Abstract

Diabetes mellitus (DM) is a chronic metabolic disorder characterized by an elevated level of blood glucose due to the absence of insulin secretion, ineffectiveness, or lack of uptake of secreted insulin in the body. The improperly diagnosed and poorly managed DM can cause severe damage to organs in the body like the nerves, eyes, heart, and kidneys. This study was aimed at investigating the effect of *Clostridium butyricum* (probiotic) with magnesium supplementation to evaluate the effect on gut microbial dysbiosis and blood glucose levels. In the laboratory, 6–8 weeks old 24 male albino rats weighing 200–250 g were given free access to water and food. Diabetes was induced using streptozotocin (60 mg/kg) in overnight fasted rats. Diabetic rats were randomly divided into four groups (*n* = 6, 6 replicates in each group). Metformin (100 mg/kg/day) with a standard basal diet was provided to control group (G_0_), *Clostridium butyricum* (1.5 × 10^5^ CFU/day) with standard basal diet was provided to treatment group (G_1_), magnesium (500 mg/kg/day) was provided to group (G_2_). *Clostridium butyricum* (1.5 × 10^5^ CFU/day) and magnesium (300 mg/kg/day) in combination with a standard basal diet was provided to group (G_3_). Blood Glucose, Magnesium blood test and microbial assay were done. Random blood glucose levels were monitored twice a week for 21 days and were represented as mean of each week. The results conclude that *Clostridium butyricum* (1.5 × 10^5^ CFU) is very effective in balancing random blood glucose levels from 206.6 ± 67.7 to 85.1 ± 3.8 (*p* = 0.006) compared to other groups (*p* > 0.005). The results of stool analysis showed that *Clostridium butyricum* as probiotic restores microbial dysbiosis as evident by the 10^5^ CFU *Clostridium butyricum* load in G_1_, which was higher than G_0_, G_2_ and G_3_ which were 10^3^ and 10^4^ CFU respectively. The findings of this study conclude that *Clostridium butyricum* supplementation improved blood glucose levels and intestinal bacterial load in type II diabetes mellitus.

## Introduction

Diabetes mellitus (DM) is a chronic metabolic disorder characterized by hyperglycemia due to either the absence of insulin secretion or an impaired hormonal response to secreted insulin in the body [1–3]. Undiagnosed and poorly managed DM can progress to severe damage to organs in the body like the nerves, eyes, heart, and kidneys [4]. DM is divided into three forms based on clinical symptoms and aetiology [5]. These categorized forms of diabetes mellitus are Type I diabetes mellitus (TIDM), Type II diabetes mellitus (T2DM) and Gestational diabetes mellitus (GDM). Dietary habits and physical activity are major contributors to human health. Lifestyle and dietary changes over the past few decades have accelerated the incidence of diabetes in adults [6]. People adopt many modifications to their diets now a day due to food availability and cheaper prices. People tend to consume more high-fat, salt-loaded, and energy-dense foods [7]. Thus, by reducing obesity and high body mass index through diet and lifestyle modifications, the burden of diabetes can be reduced [8]. Diabetes can be managed by well-balanced diet and utilization of all four types of micronutrients like macro elements, vitamins, trace elements and organic acids as they are vital for homeostasis, enzyme regulation and proper physiological functioning of the human body [9, 10]. Trace elements including cobalt, boron, chromium, copper, sulfur, iodine, zinc, molybdenum and magnesium enhance insulin action by regulating the metabolic pathways and activating insulin receptor sites [9, 11]. According to the World Health Organization (WHO), 422 million people in the world suffer from diabetes and claiming approximately 1.5 million deaths. Prevalence mostly occurs in middle and low-income countries [12, 13]. According to the National Diabetes Survey of Pakistan (NDSP 2016–17), 26.3% of the population has diabetes, which estimates 27.4 million people greater than 20 years of age have diabetes [14]. Pre-diabetes is a condition when blood sugar levels are higher than normal of a person but not high enough to be diagnosed as type 2 diabetes. In Pakistan, 16.98% of the population had type 2 diabetes and 10.91% had pre-diabetes (blood sugar level of 140 to 199 mg/dL (7.8 to 11.0 mM) 2 h after ingesting a standardized 75 gram glucose solution) [15, 16].

Probiotics usually termed as oral introduction of microorganisms in the body for certain beneficial effects. Most common probiotics used are *Lactobacillus* and *Bifidobacterium* [17–20]. For overall good health, gut microbiota should be in a balance state, any imbalance in gut micro biota causes many metabolic disorders such as insulin resistance due to production of metabolites and abnormality in utilization of nutrients [21–23]. According to previous studies there is a significant difference noted in the intestinal microbiota of diabetics and controlled samples [24]. The differences in composition of the gut microbiota may affect immunity, transit time and permeability, inflammation and energy extracted from ingested food [25, 26]. Magnesium is an important co-factor in many biochemical events, including about 300 enzymatic systems which regulate blood pressure, protein synthesis, muscle and neuron function and blood glucose regulation [27–29]. In order to treat diabetes and reduce the global burden of disease, dietary management can play a vital role [30]. The gut microbiota reside in the gastro intestinal tract [9], work together with minerals in a number of ways [31]. There are various complex mechanisms by which gut microbiota influence mineral absorption that involve several methods among minerals, microorganisms and host cells [32]. Once magnesium is up taken, intracellular Mg2 + plays a key role in regulating insulin action and insulin-mediated glucose uptake [33].

A study conducted by Ling Jia et al. 2021 in School of Food Science and Technology, Jiangnan University, Wuxi, China researched the anti-diabetic properties of *Clostridium butyricum* in mice [34]. They used 800 mg/kg/day sodium butyrate for 8 weeks and observed the lower Glycated haemoglobin (HbA1c) and fasting blood glucose when checked through oral glucose tolerance test (OGTT) and insulin tolerance test (ITT) [34]. Another study of the human model was performed by Nadja Larsen et al. (2010) in the Department of Food Science, University of Copenhagen, and Frederiksberg, Denmark. They performed this study on 36 adult men, divided into diabetic and non-diabetic male groups to identify changes in intestinal microorganisms via faces testing, investigated by tag-encoded Pyro sequencing and qPCR testing. They investigated that diabetes in adults had a strong association with compositional changes in intestinal microbiota and suggested that glucose tolerances should be considered important for diabetic treatment strategies through changes in microbiota [35]. Enterocytes synthesize claudin protein which helps for absorption of magnesium in the body [36]. In another study by Dae Jung Kim et al. (2010) in the Department of Nutrition, Gillings School of Global Public Health, and School of Medicine, the University of North Carolina conducted a 20-years follow-up study on 4,497 Americans (18–30 years of age) who had no diabetes at the start of the study. They studied the relationship of magnesium with diabetes, inflammatory markers and insulin resistance in subjects using the Coronary Artery Risk Development in Young Adults (CARDIA) study design. They found that magnesium intake had a significant inverse association with diabetes incidence in young adults. In order to determine the presence of diabetes in subjects, they use the hexokinase ultraviolet method, radioimmunoassay, Oral Glucose tolerance test, and Tosoh G-7 high performance liquid chromatography [37].

Still exact mechanisms by which gut microbiota enhance magnesium absorption and how magnesium down regulates the glucose in diabetic patient are not fully understood. The uniqueness of study is that up to now, no research work is reported from the literature for management of diabetes with magnesium supplementation and *Clostridium butyricum.*

The current research work designed to evaluate the effect of magnesium supplementation and *Clostridium butyricum* (probiotic) as probiotics which is a novel approach to cure type II diabetes mellitus as it can enhance the working efficiency of the gut microbiota and minimize the blood glucose levels.

The studies from the literature support the feasibility and benefits of using probiotic and supplementation of magnesium minimize the glucose level in diabetic and improve health.

## Materials& methods

### Induction of diabetes

All methods were performed in accordance with the relevant guidelines and regulations. The study was approved by Research Ethical Committee of University of Lahore: IRB-UOL-FAHS/822/2021. For induction of diabetes, **(**60 mg/kg) body weight of Streptozotocin (STZ) was used in overnight fasting albino rats. After Streptozotocin induction, fasting blood glucose levels of > 200 mg/dl are considered diabetic in rats [16, 38, 39]. Rats were procured from Animal Laboratory in Institute of Molecular Biology and Biotechnology (IMBB) at the University of Lahore, Pakistan.

### Rat bioassay

Male albino rats 6–8 weeks old that were 24 in number, housed in the laboratory of University Institute of Diet and Nutritional Sciences, The University of Lahore, Pakistan. Rats were kept on a standard basal diet for 1 week for acclimatization purposes with controlled conditions like temperature (23 ± 2 ^0^C) and relative humidity (55%), along with a 12-hour light-dark period, and these conditions were followed throughout the trial. The rats feeding time was 2:00 PM daily and blood sampling time was at 5:00 PM. The fasting conditions were maintained before blood sampling and were consistent across all groups throughout the research work. Glucometer was calibrated and operated according to the instructions given in the operating manual of the glucometer. A total of 24 rats were randomly divided into four groups (n = 6). G_0_ had 6 rats, which were diabetic control groups and fed a standard basal diet and metformin at (100 mg/kg/day) [40, 41]. The randomization was done using “random number generator”. The source of Closmtridium butyricum used in the study was from “The Searle Company Limited” and brand name is “Gut Care” available in Pakistan. G_1_ had six diabetic rats fed probiotic *Clostridium butyricum* (1.5 × 10^5^ CFU/day) with a standard basal diet. G_2_ had six diabetic rats fed magnesium (500 mg/kg) with a standard basal diet. G_3_ had six diabetic rats given a combined probiotic (1.5 × 10^5^ CFU/day) and magnesium (300 mg/kg) with a standard basal diet [42, 43]. The fed was procured from “National feed company”, with brand name “Broiler pre starter crumbs”, Feed no. 14”. The producer country is Pakistan for this feed.

After the induction of diabetes, the values taken at very first day were called pre-treatment. The values taken after the completion of treatment i.e. at the end of treatment were called post-treatment values. The rats were euthanized using the cervical dislocation technique [44].

### Glucose assay

Random and fasting blood glucose levels were measured through the Glucometer device [15].

### Magnesium blood test

Blood magnesium levels were measured by the Magnesium Assay Kit manufactured by Randox Laboratories, United States of America. A calmagite dye in the kit forms a colored complex specifically with magnesium. The intensity of the color, measured at 500 nm, is directly proportional to the magnesium concentration in the sample [28].

### Microbial assay

Feces were collected from rat cages [45]. 30 g of samples were diluted with 150 mL of sterile saline, homogenized in a standard blender, and filtered through gauze three times [46]. It was centrifuged at 6,000 rpm for 15 min, re-suspended in 150 mL of fresh sterile saline, and transferred directly to blood agar at a room temperature of 30 °C in an anaerobic incubator [47]. After incubation at 37 ^0^C for 48 to 72 h, colonies were identified on the basis of colony morphology [48].

### Statistical analysis

Data analysis was done using SPSS version 20, considering *p*-value < 0.05 as level of significance. Quantitative variables like weight, blood glucose levels were represented as Mean ± SD. Differences in pre-treatment and post treatment variables like fasting and random blood glucose levels were analyzed using paired sample T-test.

## Results

**Table 1:** Pre and post treatment Mean ± SD values of random blood glucose levels of Albino Rats induced diabetic with streptozotocin

Groups	Pre Treatment Mean ± SD	Post Treatment Mean ± SD	Percent change (%)	*P* value
**Week 1**				
G_0_	206.6 ± 67.7	246.1 ± 17.1	19.1	0.203
G_1_	206.6 ± 67.7	189.3 ± 32.6	8.4	0.431
G_2_	231.7 ± 101.3	255.2 ± 34.3	10.1	0.534
G_3_	126 ± 7.07	213 ± 7.07	69.0	0.429
**Week 2**				
G_0_	206.6 ± 67.7	260.1 ± 13.3	25.9	0.024
G_1_	206.6 ± 67.7	142.3 ± 10.9	31.1	0.052
G_2_	231.7 ± 101.3	328.5 ± 28.29	41.8	0.077
G_3_	126 ± 7.07	348.7 ± 7.4	176.7	0.001
**Week 3**				
G_0_	206.6 ± 67.7	370 ± 22.3	79.1	0.005
G_1_	206.6 ± 67.7	85.1 ± 3.8	58.8	0.006
G_2_	231.7 ± 101.3	354.2 ± 17.6	52.9	0.061
G_3_	126 ± 7.07	288.5 ± 7.07	129.0	0.008

G_0_: Metformin (100 mg/kg/day) with a standard basal diet

G_1_: *Clostridium butyricum* (probiotic) (1.5 × 10^5^ CFU/day) with standard basal diet

G_2_: Magnesium (500 mg/kg/day)

G_3_:*Clostridium butyricum* (probiotic) (1.5 × 10^5^ CFU/day) and magnesium (300 mg/kg/day) in combination with a standard basal diet

Table 1 shows the random blood glucose levels of rats of all groups. Paired sample t-test was performed to observe the changes of treatment. Results are represented as mean value with standard deviation. In control group G_0_, during week 1st there was no significant increase in random blood glucose level by 19.1%, during week 2nd significant increase in random blood levels by 25.9% and at the end of week 3rd significant elevated random blood glucose levels by 79.1%. In G_1_, during week 1st no significant reduction in random blood glucose levels were recorded and it was 8.4%, during week 2nd significant reduction in blood glucose levels were observed as decrease in random blood glucose levels by 31.1%. At the end of week 3rd there was significant decrease in random blood glucose by 58.8%. In G_2_, during week 1st there was no significant difference in random blood glucose levels (RBGL) by increase by 10.1%, while in week 2nd and 3rd there was no significant difference in blood glucose levels by increase in RBGL by 41.8% and 52.9% respectively. In G_3_, there was no significant difference observed during week 1st as increase in RBGL by 69%, while significant difference observed in week 2nd by increase in RBGL by 176.7% and 3rd by elevated RBGL by 129% respectively. The results showed that G_1_ had significant reduction in RBGL by 8.4%, 31.1% and 58.8% respectively during the week wise treatment.

**Table 2:** Pre and post treatment Mean ± SD values of fasting blood glucose levels in streptozotocin induced diabetic rats

Group	Pre-Treatment Mean ± SD	Post-Treatment Mean ± SD	Percent change (%)	*P*-value
G_0_	142.6 ± 12.2	261.6 ± 17.9	83.5	0.000
G_1_	101 ± 2.68	72.33 ± 16.8	28.4	0.004
G_2_	115.0 ± 2.3	232 ± 124.7	101.7	0.152
G_3_	140.5 ± 6.36	239.5 ± 7.7	70.5	0.006

G_0_: Metformin (100 mg/kg/day) with a standard basal diet

G_1_: *Clostridium butyricum* (probiotic) (1.5 × 10^5^ CFU/day) with standard basal diet

G_2_: Magnesium (500 mg/kg/day)

G_3_: *Clostridium butyricum* (probiotic) (1.5 × 10^5^ CFU/day) and magnesium (300 mg/kg/day) in combination with a standard basal diet

Table 2 shows the mean values of fasting blood glucose levels for all groups’ pre and post treatment. Results are represented as mean values with standard deviation. In G_0_ there was significant increase in fasting blood glucose levels(FBGL) by 83.5%, in G_1_ there was signification reduction in fasting blood glucose levels (FBGL) by 28.4%, in G_2_ there was increase in fasting blood glucose levels(FBGL) by 101.7%, while in G_3_ there was increase in FBGL by 70.5%. These results showed that G_1_ had significant reduction in fasting blood glucose levels (FBGL) by 28.4% at the end of treatment as shown in the Table 2.

**Table 3:** Pre and post-treatment Mean ± SD values of serum magnesium in streptozotocin-induced diabetic rats

Group	Pre Treatment Mean ± SD	Post Treatment Mean ± SD	Percent change (%)	*P* value
G_0_	0.24 ± 0.00	0.27 ± 0.11	11.1	0.256
G_1_	0.28 ± 0.10	0.40 ± 0.15	30.0	0.116
G_2_	0.26 ± 0.12	0.38 ± 0.05	31.6	0.049
G_3_	0.27 ± 0.04	0.45 ± 0.15	40.0	0.442

G_0_: Metformin (100 mg/kg/day) with a standard basal diet

G_1_: *Clostridium butyricum* (probiotic) (1.5 × 10^5^ CFU/day) with standard basal diet

G_2_: Magnesium (500 mg/kg/day)

G_3_: *Clostridium butyricum* (probiotic) (1.5 × 10^5^ CFU/day) and magnesium (300 mg/kg/day) in combination with a standard basal diet

Table 3 shows the mean values of serum magnesium levels for all groups’ pre- and post-treatment. Results are represented as a mean value with a standard deviation. G_0_ showed no significant increase in serum magnesium levels by 11.1%, and G_1_ expressed no significant increase in serum magnesium levels by 30.0%. G_2_ showed a significant increase in serum magnesium levels by 31.1%, while in G_3_, there was no significant increase in serum magnesium levels by 40.0%. These results showed that there was a significant increase in serum magnesium levels only in the magnesium-fed group (G_2_), but all groups remained hypomagnesaemia as mentioned in the Table 3.

**Fig. 1:**
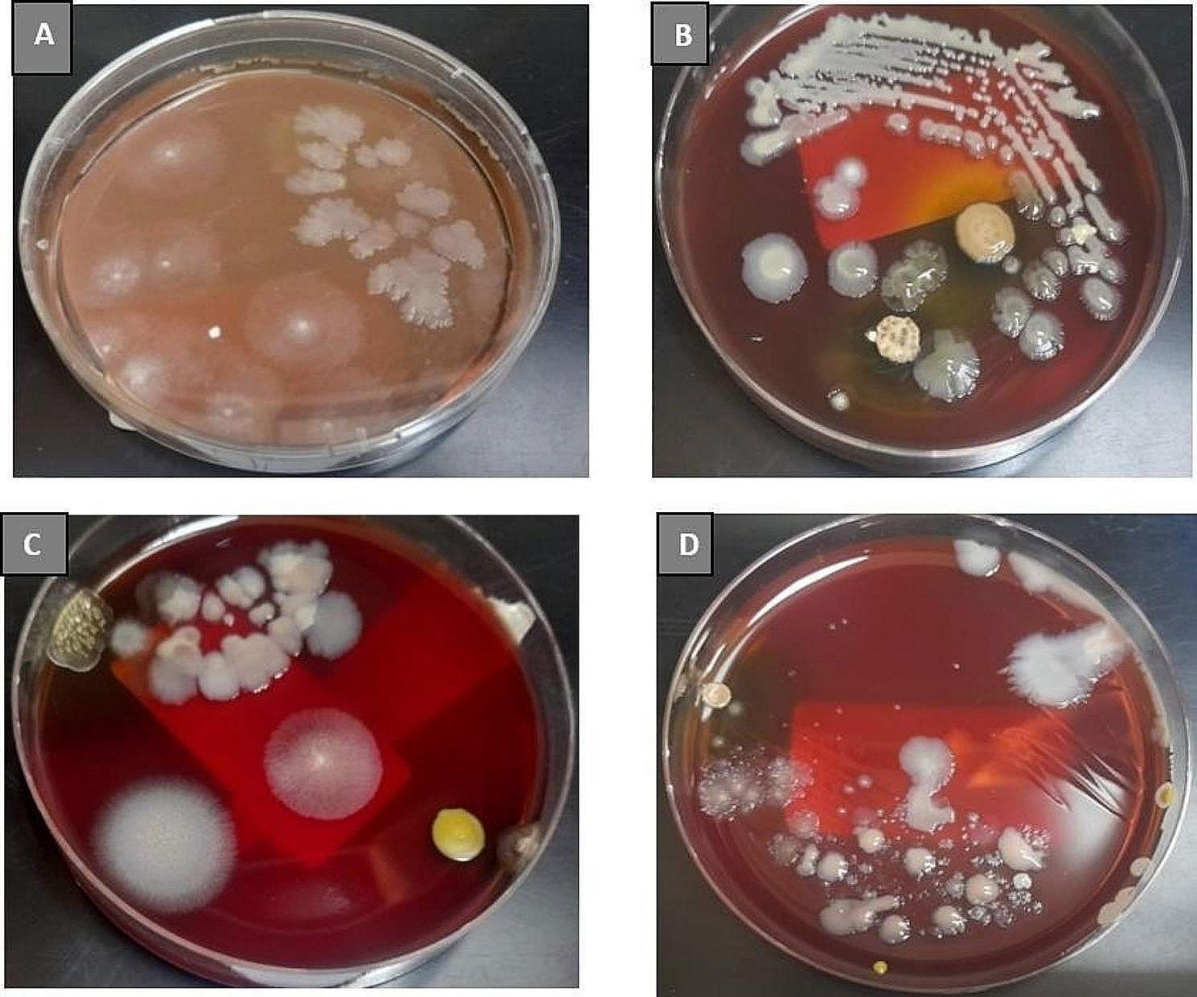
*Clostridium butyricum* (probiotic) load in rat fecal samples. (**A**) 10^3^ CFU of *Clostridium butyricum* (probiotic) load in control group G0. (**B**) 10^5^ CFU of *Clostridium butyricum* (probiotic) in group G1. (**C**) 10^3^ CFU of *Clostridium butyricum* (probiotic) load in group G2. (**D**) 10^4^ CFU load of *Clostridium butyricumin* (probiotic) G_3_

*Clostridium butyricum* alone and along with magnesium supplements restores gut dysbiosis in streptozotocin-induced diabetic rats. Maximum bacterial count 10^5^ CFU was observed in G_1_ followed by G_3_ (10^4^ CFU) > G_2_ (10^3^ CFU).

## Discussion

Diabetes mellitus is commonly associated with Mg^2+^ deficiency. In type II diabetes mellitus one of the most important causes of Mg^2+^ deficiency is less intakes or more excretion via urine. It is a fact that most diabetic individuals have low Mg^2+^ in blood as compared with non-diabetic [49, 50]. Magnesium is involved in the regulation of insulin signaling, phosphorylation of insulin receptor kinase, post receptorial action of insulin and also in insulin-mediated cellular glucose uptake [50, 51]. Probiotics have a critical role in managing diabetes by affecting random blood glucose levels and improving gut function [52]. Supplementation of the strain *Clostridium butyricum* CGMCC0313.1 replenished the liver functioning, boosted gut microbiota in diabetic. Changes in gut microbiota were associated with higher peroxisome proliferator–activated receptor-γ, insulin signaling molecules and increased butyrate production. *Clostridium butyricum* improved diabetic markers like glucose tolerance, fasting glucose, insulin tolerance, Glucagon-like peptide-1 receptor and insulin secretion [34]. The results of our study suggest that the used strain of *Clostridium butyricum* may act in a similar way as a beneficial probiotic for the prevention and treatment of diabetes. A study designed the placebo trial to observe the effect of magnesium on diabetes. 300 mg magnesium per day as magnesium sulphate for 3 months was given as compared to placebo, and they observed a positive effect of magnesium supplementation on random blood glucose levels from (239.1 ± 74.75 to 189.1 ± 60 mg/dL versus 246.4 ± 97.37 to 247.8 ± 86.74 mg/dL, *p* < 0.01) [52], while contrary to our study, it showed no effect of magnesium supplementation on random blood glucose levels from 231.7 ± 101.3 to 354.2 ± 17.6 as a *P* value 0.06. The positive effect of magnesium on RBG levels could not be observed due to the shorter duration of treatment in this study or secondly the use of magnesium oxide instead of magnesium sulphate [53]. Hani Al Salami et al., in 2008, experimented with this study to evaluate the effect of probiotics with gliclazide in diabetic rats, 40 rats were divided into 4 groups, and probiotic 75 mg/kg was given to rats. They observed random blood glucose levels decreasing by 2 folds (*P* < 0.01) in diabetic rats [54]. In this study, probiotic *Clostridium butyricum* also decreased RBG levels from 206.6 ± 67.7 to 85.1 ± 3.8 with a *P* value of 0.006. As *Clostridium butyricum* produced short chain fatty acids and butyrate producing bacteria which improves gut homeostasis and helps in controlling random blood glucose levels especially in diabetes [55]. Elevated fasting blood glucose level is characteristic sign of diabetes. Probiotic improves fasting blood glucose levels in diabetes [56]. A study was performed to access the hypoglycemic effect of *Clostridium butyricum* in rat trial where animals were divided into 2 groups and given *Clostridium butyricum* (2.5 × 10^8^) for 45 weeks, and observed significant results (*P* < 0.0172) in FBG levels. They also observed that *Clostridium butyricum* feeding at an early age delayed the onset of diabetes in rats [34]. The findings of this study also showed impressive effects in lowering FBG levels by the use of *Clostridium butyricum*. The results indicate that G_0_ on a normal diet with metformin had no major impact on FBG levels from 142.6 ± 12.2 to 261.6 ± 17.9 (*P* value = 0.000), while G_1_ with *Clostridium butyricum* treatment showed significant effects in lowering FBG levels from 101 ± 2.68 to 72.33 ± 16.8 (*P* value = 0.004). Short chain fatty acids regulate blood glucose through hormones. One of the glucose transporters GLUT4 allows glucose to enter the cells in skeletal muscle cells. Short chain fatty acids increase the expression receptors of GLUT4 and translocate those receptors to the cells plasma membranes, enhancing the absorption of more glucose by cells [57].

G_2_ with magnesium treatment also had no such good impact on FBG levels, such as 115.0 ± 2.3 to 232 ± 124.7 values showing an increasing trend as *P* value = 0.152, which shows that magnesium had no effect in lowering blood glucose levels, instead of it magnesium in this study had no positive effects, magnesium solely caused bloating and constipation in rats. While G3, which was magnesium + probiotic, showed a significant increasing trend in FBG levels from 140.5 ± 6.36 to 239.5 ± 7.7 as *P* value = 0.006. In this study, it was hypothesized that solely magnesium supplementation through oral diet would reduce hyperglycemia, however the results showed that magnesium supplementation had no effect on lowering hyperglycemia, instead, it caused intestinal inflammation and paralytic ileus in rats, and 66.6% of rats died due to magnesium toxicity and hyperglycemia in this group [58]. G_0_ showed a significant increase in serum magnesium levels from 0.24 ± 0.00 mg/dL to 0.27 mg/dL ± 0.11 observed as *P* = 0.256; G_1_ expressed no significant increase in serum magnesium levels from 0.28 ± 0.10 mg/dL to 0.40 ± 0.15 mg/dL observed as *P* = 0.116. G_2_ showed a significant increase in serum magnesium levels from 0.26 ± 0.12 mg/dL to 0.38 ± 0.05 mg/dL observed as *P* = 0.049, while in G_3_ there was no significant increase in serum magnesium levels from 0.27 ± 0.04 mg/dL to 0.45 ± 0.15 mg/dL observed as *P* = 0.442. These results showed that there was a significant increase in serum magnesium levels only in the magnesium-fed group (G_2_), but all groups remained hypomagnesaemia. Hypomagnesemia is reported in patients with type II diabetes due to less intake and more excretion from the body due to frequent urination. Supplementation of magnesium results in increase of intracellular Mg^2+^ that plays a key role in regulation of insulin action [50, 51]. A study designed by Naila Masood et al. in 2009 observed the same effect that magnesium concentration had no significant difference in diabetes and the control group from 22.67 ± 24.5 mg/dL to 18.3 ± 3.4 mg/dL, *P* = 0.26 [59]. The positive effect of magnesium in controlling diabetes, which is described in various investigations, may not be observed in this study due to magnesium being given in the form of magnesium oxide, which caused constipation and bloating. As magnesium response is dose-dependent, the high dose of magnesium oxide in this study caused the problems stated above.

The use of Probiotic supplementation in diabetic control has many advantages. Probiotics improve gut flora, manage gut homeostasis, and reduce intestinal inflammation to improve insulin sensitivity [60]. Induction of *Clostridium butyricum* via oral administration in rats improved *Clostridium butyricum* load in gut microbiota composition. Ling Jia et al. in 2017 observed the same effect by conducting a study trail in which they divided rats into 2 groups and gave one group *Clostridium butyricum* for 5 weeks and the second group the same probiotic for 13 weeks, and they observed a significant change in the composition of *Clostridium butyricum* (*P* = 0.385) [34]. In this study, *Clostridium butyricum* changes were also observed via microbial analysis. Gut homeostasis plays a major role in managing metabolic diseases. The changes in the composition of *Clostridium butyricum* in each group indicate that G1reported from Fig. 1 as of petri plate B, which had a greater number of *Clostridium butyricum* 10^5^ CFU/mL than other groups, had a significant impact on lowering random blood glucose levels (*P* value = 0.006), and an increase in weight was also observed in this group from 246.6 ± 8.6 to 258 ± 11.6 (*P* value = 0.001). The observations and results from G_0_ 10^3^ CFU/mL, from petri plate A indicates that a lower number of *Clostridium butyricum* was associated with a decrease in weight from 261.0 ± 8.08 to 197.7 ± 26.8 such as (*P* value = 0.036) and random blood glucose levels from 206.6 ± 67.7 to 370 ± 22.3 as the (*P* value = 0.005). In G2, from petri plate C, the same trend of *Clostridium butyricum* was observed at 10^3^ CFU/mL, which was less than as compared to G1 and results indicate that there was a significant decrease in weight from 251.3 ± 12.1 to 186.6 ± 13.8 as (*P* value of 0.000), and the same trend of a lower load of *Clostridium butyricum* was observed in random blood glucose levels. There was increase in random blood glucose levels from 231.7 ± 101.3 to 328.5 ± 28.29 as (*P* value = 0.077), the same as the fasting blood glucose levels of G_2_ from 115.0 ± 2.3 to 232 ± 124.7 as (*P* value = 0.152). Group G3, petri plate D, Fig. 1, had a same relation of *Clostridium butyricum* load in fecal samples, observed as 10^4^CFU/ml, which was greater than G2 and G1 due to supplementation of *Clostridium butyricum* in combination with magnesium. Significant weight change occurred as 266 ± 7.07 to 181.2 ± 13.7 as (*P* value = 0.036), and random blood glucose levels as 126 ± 7.07 to 288.5 ± 7.07 as (*P* values = 0.008). Fasting blood glucose levels were observed 140.5 ± 6.36 to 239.5 ± 7.7 with *P* value = 0.006. These results showed that *Clostridium butyricum* has major role in diabetes management as this probiotic produces short chain fatty acids which regulate intestinal homeostasis and help regulate blood glucose levels and increase growth performance [55].

## Conclusion

The findings of this study reveal that, contrary to many other studies, magnesium didn’t show effective results in controlling blood glucose levels alone. However, *Clostridium butyricum* (1.5 × 10^5^ CFU)-fed rats G_1_ showed great improvement in fasting and random blood glucose levels as compared to other groups, which were diabetic and magnesium-fed groups. *Clostridium butyricum* improved gut health, maintained the homeostasis well and metabolism of the gut micro biota which was proved by microbial analysis of a *Clostridium butyricum* load of 105 CFU in stool samples of rats followed by magnesium and *Clostridium butyricum* combination G_3,_ and G_2_. Further studies are needed to evaluate the effect of magnesium oxide and the dose of magnesium in combination with *Clostridium butyricum* in diabetes for better and safer results. There should be also study only with magnesium using doses and also separately using magnesium oxide. A study is also need when using combine different doses of magnesium and clostridium butyricum to evaluate the effect on hyperglycemia.

## Limitations/challenges

We used Spruge dully albino rats and in study comparison was done with varieties of albino rats.

We used feed with brand name “Broiler pre starter crumbs”, Feed no. 14”. Company name “National feed company”, the producer country is Pakistan for this feed.

All the experimental work was carried out in “The University of Lahore”, Pakistan. That has different environment with other countries.

## Data Availability

Not applicable.
